# Supplementary material for: New Color-Patterned Species of Microtendipes Kieffer, 1913 (Diptera: Chironomidae) and a Deep Intraspecific Divergence of Species by DNA Barcodes
Source: Insects. 2023 Feb 24;14(3):227. doi: 10.3390/insects14030227 (PMC10054112; doi:10.3390/insects14030227)
Supplement: Supplementary file 1 [file insects-14-00227-s001.zip › Figure S3.pdf]

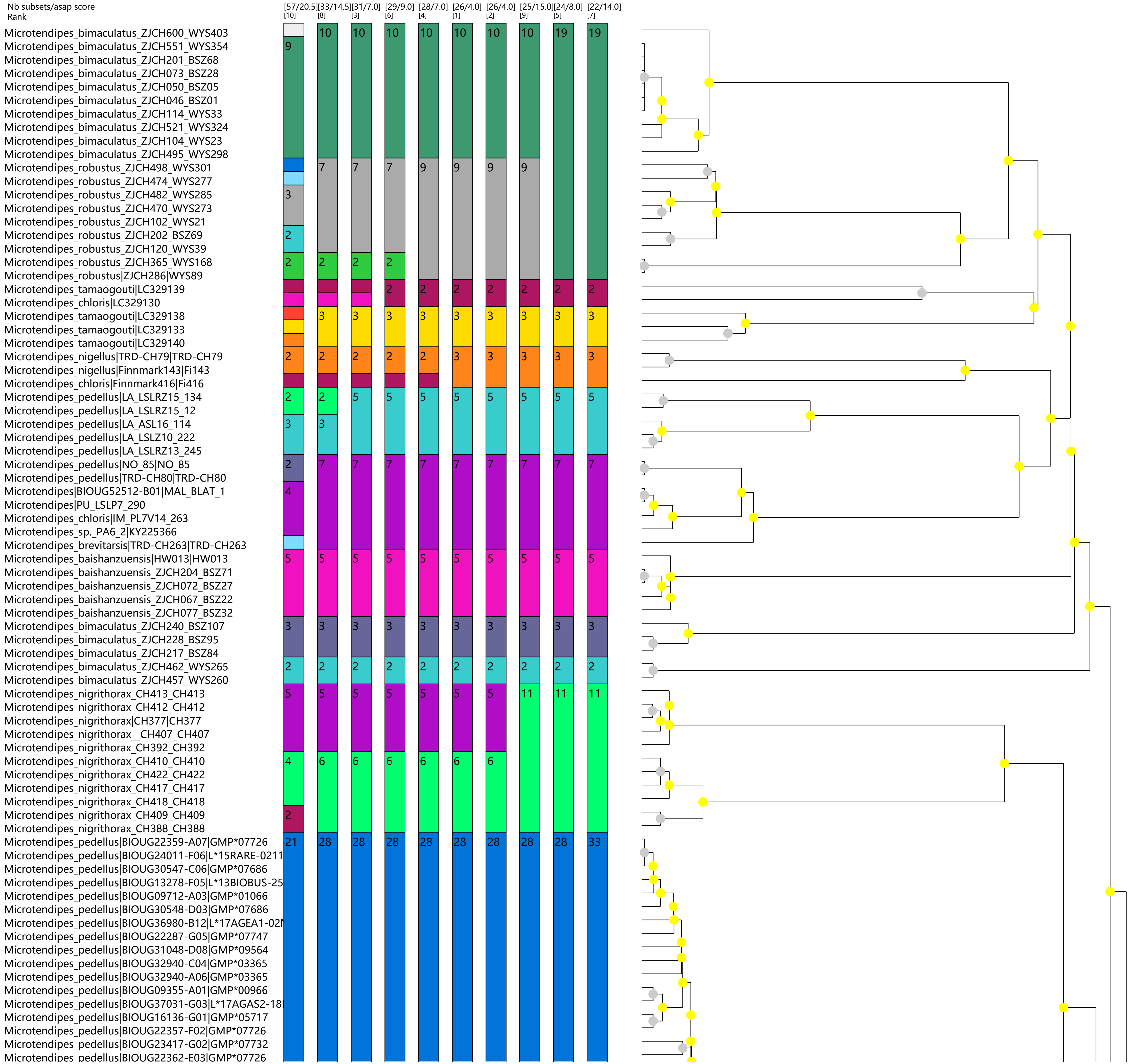
Nb subsets/asap score
Rank
[57/20.5] [10]
[33/14.5] [8]
[31/7.0] [3]
[29/9.0] [6]
[28/7.0] [4]
[26/4.0] [1]
[26/4.0] [2]
[25/15.0] [9]
[24/8.0] [5]
[22/14.0] [7]
Microtendipes_bimaculatus_ZJCH600_WYS403
Microtendipes_bimaculatus_ZJCH551_WYS354
Microtendipes_bimaculatus_ZJCH201_BSZ68
Microtendipes_bimaculatus_ZJCH073_BSZ28
Microtendipes_bimaculatus_ZJCH050_BSZ05
Microtendipes_bimaculatus_ZJCH046_BSZ01
Microtendipes_bimaculatus_ZJCH114_WYS33
Microtendipes_bimaculatus_ZJCH521_WYS324
Microtendipes_bimaculatus_ZJCH104_WYS23
Microtendipes_bimaculatus_ZJCH495_WYS298
Microtendipes_robustus_ZJCH498_WYS301
Microtendipes_robustus_ZJCH474_WYS277
Microtendipes_robustus_ZJCH482_WYS285
Microtendipes_robustus_ZJCH470_WYS273
Microtendipes_robustus_ZJCH102_WYS21
Microtendipes_robustus_ZJCH202_BSZ69
Microtendipes_robustus_ZJCH120_WYS39
Microtendipes_robustus_ZJCH365_WYS168
Microtendipes_robustus|ZJCH286|WYS89
Microtendipes_tamaogouti|LC329139
Microtendipes_chloris|LC329130
Microtendipes_tamaogouti|LC329138
Microtendipes_tamaogouti|LC329133
Microtendipes_tamaogouti|LC329140
Microtendipes_nigellus|TRD-CH79|TRD-CH79
Microtendipes_nigellus|Finnmark143|Fi143
Microtendipes_chloris|Finnmark416|Fi416
Microtendipes_pedellus|LA_LSLRZ15_134
Microtendipes_pedellus|LA_LSLRZ15_12
Microtendipes_pedellus|LA_ASL16_114
Microtendipes_pedellus|LA_LSLZ10_222
Microtendipes_pedellus|LA_LSLRZ13_245
Microtendipes_pedellus|NO_85|NO_85
Microtendipes_pedellus|TRD-CH80|TRD-CH80
Microtendipes|BIOUG52512-B01|MAL_BLAT_1
Microtendipes|PU_LSLP7_290
Microtendipes_chloris|IM_PL7V14_263
Microtendipes_sp._PA6_2|KY225366
Microtendipes_brevitarsis|TRD-CH263|TRD-CH263
Microtendipes_baishanzuensis|HW013|HW013
Microtendipes_baishanzuensis_ZJCH204_BSZ71
Microtendipes_baishanzuensis_ZJCH072_BSZ27
Microtendipes_baishanzuensis_ZJCH067_BSZ22
Microtendipes_baishanzuensis_ZJCH077_BSZ32
Microtendipes_bimaculatus_ZJCH240_BSZ107
Microtendipes_bimaculatus_ZJCH228_BSZ95
Microtendipes_bimaculatus_ZJCH217_BSZ84
Microtendipes_bimaculatus_ZJCH462_WYS265
Microtendipes_bimaculatus_ZJCH457_WYS260
Microtendipes_nigrithorax_CH413_CH413
Microtendipes_nigrithorax_CH412_CH412
Microtendipes_nigrithorax|CH377|CH377
Microtendipes_nigrithorax__CH407_CH407
Microtendipes_nigrithorax_CH392_CH392
Microtendipes_nigrithorax_CH410_CH410
Microtendipes_nigrithorax_CH422_CH422
Microtendipes_nigrithorax_CH417_CH417
Microtendipes_nigrithorax_CH418_CH418
Microtendipes_nigrithorax_CH409_CH409
Microtendipes_nigrithorax_CH388_CH388
Microtendipes_pedellus|BIOUG22359-A07|GMP*07726
Microtendipes_pedellus|BIOUG24011-F06|L*15RARE-0211
Microtendipes_pedellus|BIOUG30547-C06|GMP*07686
Microtendipes_pedellus|BIOUG13278-F05|L*13BIOBUS-25
Microtendipes_pedellus|BIOUG09712-A03|GMP*01066
Microtendipes_pedellus|BIOUG30548-D03|GMP*07686
Microtendipes_pedellus|BIOUG36980-B12|L*17AGEA1-02
Microtendipes_pedellus|BIOUG22287-G05|GMP*07747
Microtendipes_pedellus|BIOUG31048-D08|GMP*09564
Microtendipes_pedellus|BIOUG32940-C04|GMP*03365
Microtendipes_pedellus|BIOUG32940-A06|GMP*03365
Microtendipes_pedellus|BIOUG09355-A01|GMP*00966
Microtendipes_pedellus|BIOUG37031-G03|L*17AGAS2-18
Microtendipes_pedellus|BIOUG16136-G01|GMP*05717
Microtendipes_pedellus|BIOUG22357-F02|GMP*07726
Microtendipes_pedellus|BIOUG23417-G02|GMP*07732
Microtendipes_pedellus|BIOUG22362-E03|GMP*07726

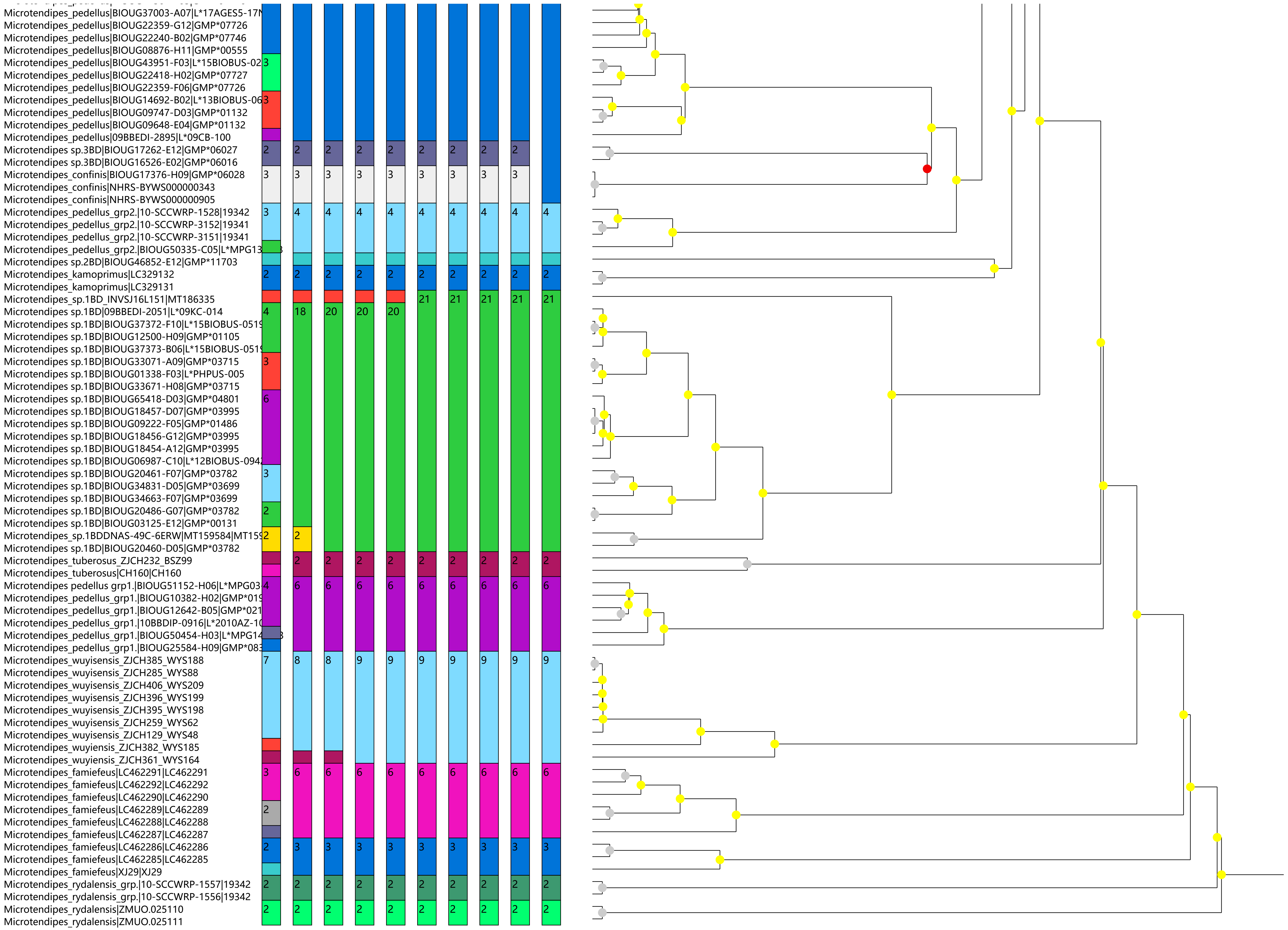
Microtendipes_pedellus|BIOUG37003-A07|L*17AGES5-17N
Microtendipes_pedellus|BIOUG22359-G12|GMP*07726
Microtendipes_pedellus|BIOUG22240-B02|GMP*07746
Microtendipes_pedellus|BIOUG08876-H11|GMP*00555
Microtendipes_pedellus|BIOUG43951-F03|L*15BIOBUS-02
Microtendipes_pedellus|BIOUG22418-H02|GMP*07727
Microtendipes_pedellus|BIOUG22359-F06|GMP*07726
Microtendipes_pedellus|BIOUG14692-B02|L*13BIOBUS-06
Microtendipes_pedellus|BIOUG09747-D03|GMP*01132
Microtendipes_pedellus|BIOUG09648-E04|GMP*01132
Microtendipes_pedellus|09BBEDI-2895|L*09CB-100
Microtendipes sp.3BD|BIOUG17262-E12|GMP*06027
Microtendipes sp.3BD|BIOUG16526-E02|GMP*06016
Microtendipes_confinis|BIOUG17376-H09|GMP*06028
Microtendipes_confinis|NHRS-BYWS000000343
Microtendipes_confinis|NHRS-BYWS000000905
Microtendipes_pedellus_grp2.|10-SCCWRP-1528|19342
Microtendipes_pedellus_grp2.|10-SCCWRP-3152|19341
Microtendipes_pedellus_grp2.|10-SCCWRP-3151|19341
Microtendipes_pedellus_grp2.|BIOUG50335-C05|L*MPG13
Microtendipes sp.2BD|BIOUG46852-E12|GMP*11703
Microtendipes_kamoprimus|LC329132
Microtendipes_kamoprimus|LC329131
Microtendipes_sp.1BD_INVSJ16L151|MT186335
Microtendipes sp.1BD|09BBEDI-2051|L*09KC-014
Microtendipes sp.1BD|BIOUG37372-F10|L*15BIOBUS-0519
Microtendipes sp.1BD|BIOUG12500-H09|GMP*01105
Microtendipes sp.1BD|BIOUG37373-B06|L*15BIOBUS-0519
Microtendipes sp.1BD|BIOUG33071-A09|GMP*03715
Microtendipes sp.1BD|BIOUG01338-F03|L*PHPUS-005
Microtendipes sp.1BD|BIOUG33671-H08|GMP*03715
Microtendipes sp.1BD|BIOUG65418-D03|GMP*04801
Microtendipes sp.1BD|BIOUG18457-D07|GMP*03995
Microtendipes sp.1BD|BIOUG09222-F05|GMP*01486
Microtendipes sp.1BD|BIOUG18456-G12|GMP*03995
Microtendipes sp.1BD|BIOUG18454-A12|GMP*03995
Microtendipes sp.1BD|BIOUG06987-C10|L*12BIOBUS-094
Microtendipes sp.1BD|BIOUG20461-F07|GMP*03782
Microtendipes sp.1BD|BIOUG34831-D05|GMP*03699
Microtendipes sp.1BD|BIOUG34663-F07|GMP*03699
Microtendipes sp.1BD|BIOUG20486-G07|GMP*03782
Microtendipes sp.1BD|BIOUG03125-E12|GMP*00131
Microtendipes_sp.1BDDNAS-49C-6ERW|MT159584|MT159
Microtendipes sp.1BD|BIOUG20460-D05|GMP*03782
Microtendipes_tuberosus_ZJCH232_BSZ99
Microtendipes_tuberosus|CH160|CH160
Microtendipes pedellus grp1.|BIOUG51152-H06|L*MPG03
Microtendipes_pedellus_grp1.|BIOUG10382-H02|GMP*01
Microtendipes_pedellus_grp1.|BIOUG12642-B05|GMP*021
Microtendipes_pedellus_grp1.|10BBDIP-0916|L*2010AZ-1
Microtendipes_pedellus_grp1.|BIOUG50454-H03|L*MPG14
Microtendipes_pedellus_grp1.|BIOUG25584-H09|GMP*08
Microtendipes_wuyisensis_ZJCH385_WYS188
Microtendipes_wuyisensis_ZJCH285_WYS88
Microtendipes_wuyisensis_ZJCH406_WYS209
Microtendipes_wuyisensis_ZJCH396_WYS199
Microtendipes_wuyisensis_ZJCH395_WYS198
Microtendipes_wuyisensis_ZJCH259_WYS62
Microtendipes_wuyisensis_ZJCH129_WYS48
Microtendipes_wuyiensis_ZJCH382_WYS185
Microtendipes_wuyiensis_ZJCH361_WYS164
Microtendipes_famiefeus|LC462291|LC462291
Microtendipes_famiefeus|LC462292|LC462292
Microtendipes_famiefeus|LC462290|LC462290
Microtendipes_famiefeus|LC462289|LC462289
Microtendipes_famiefeus|LC462288|LC462288
Microtendipes_famiefeus|LC462287|LC462287
Microtendipes_famiefeus|LC462286|LC462286
Microtendipes_famiefeus|LC462285|LC462285
Microtendipes_famiefeus|XJ29|XJ29
Microtendipes_rydalensis_grp.|10-SCCWRP-1557|19342
Microtendipes_rydalensis_grp.|10-SCCWRP-1556|19342
Microtendipes_rydalensis|ZMUO.025110
Microtendipes_rydalensis|ZMUO.025111
