# Supplementary figures and images for: New Color-Patterned Species of Microtendipes Kieffer, 1913 (Diptera: Chironomidae) and a Deep Intraspecific Divergence of Species by DNA Barcodes
Source: Insects. 2023 Feb 24;14(3):227. doi: 10.3390/insects14030227 (PMC10054112; doi:10.3390/insects14030227)

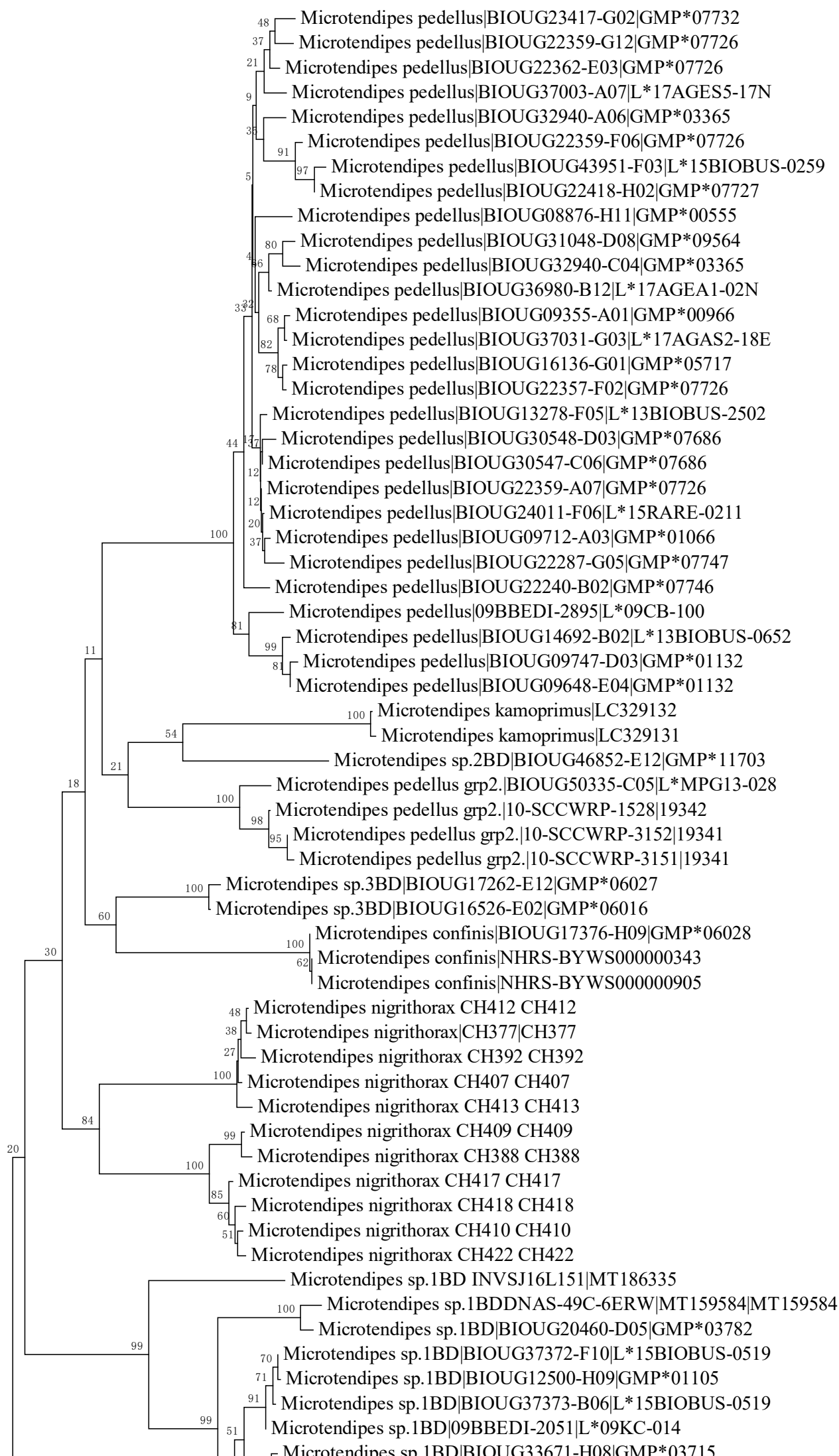

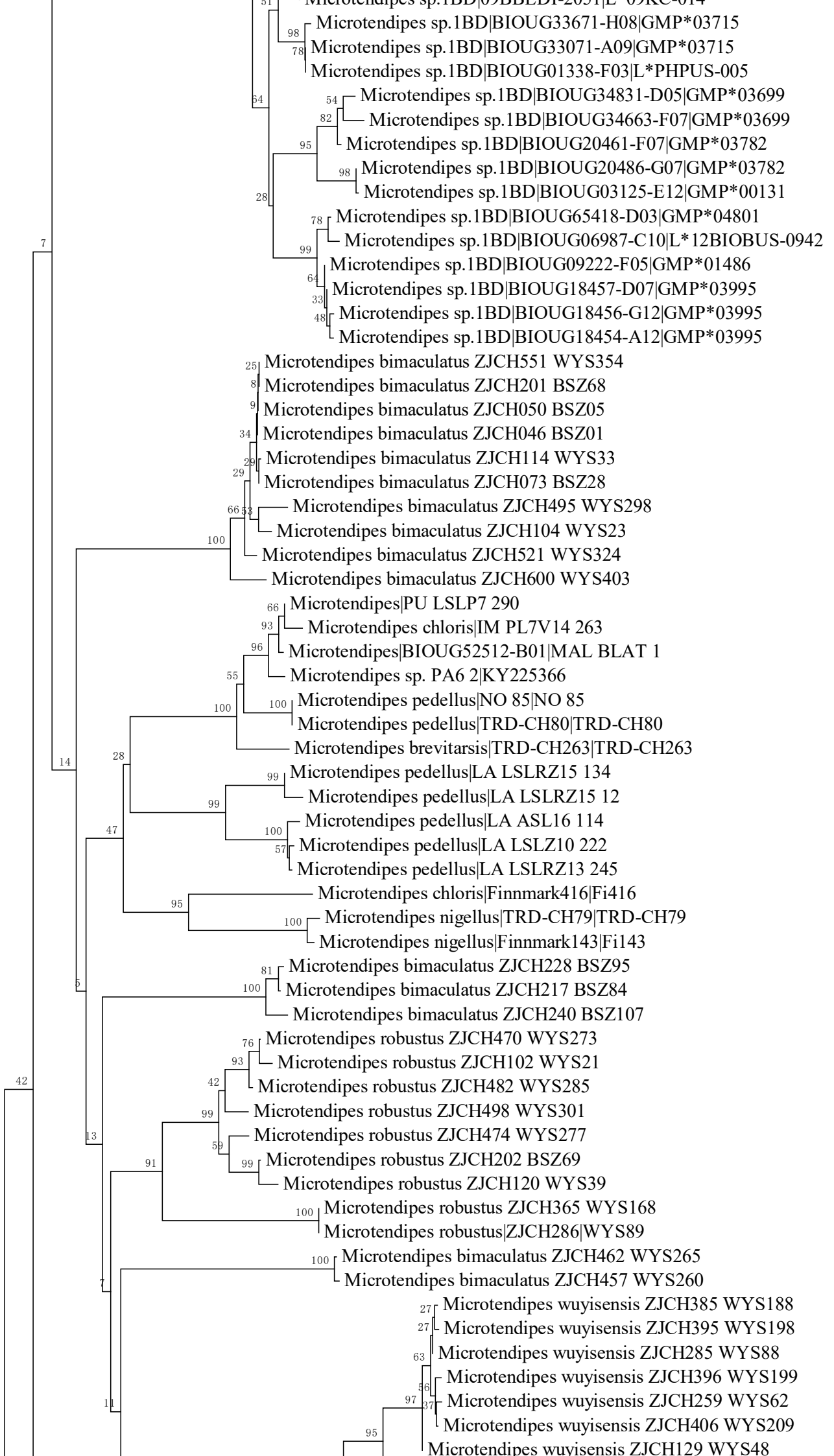

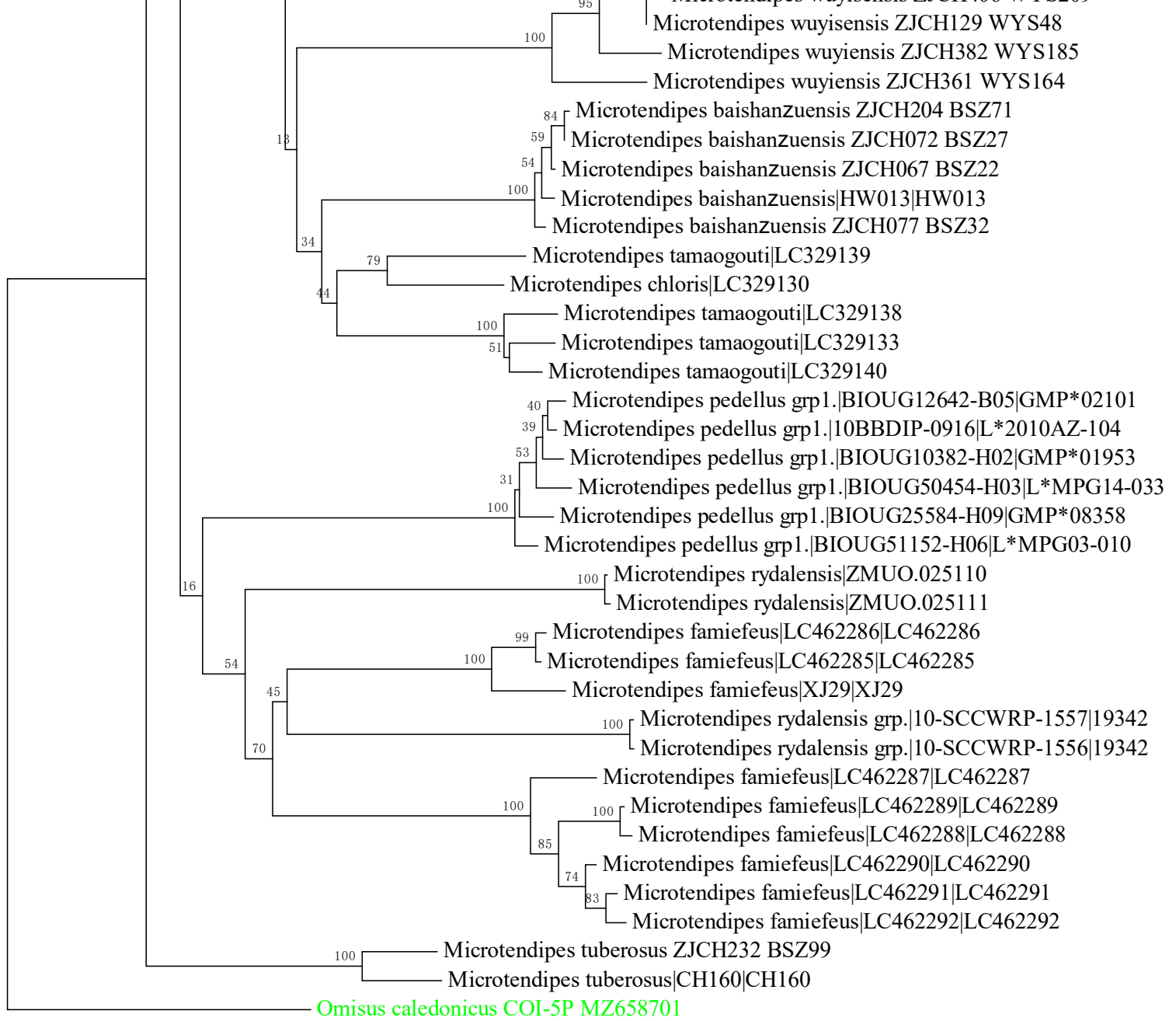

0.0100

Supplement: Supplementary file 1 [file insects-14-00227-s001.zip › Figure S1.pdf]

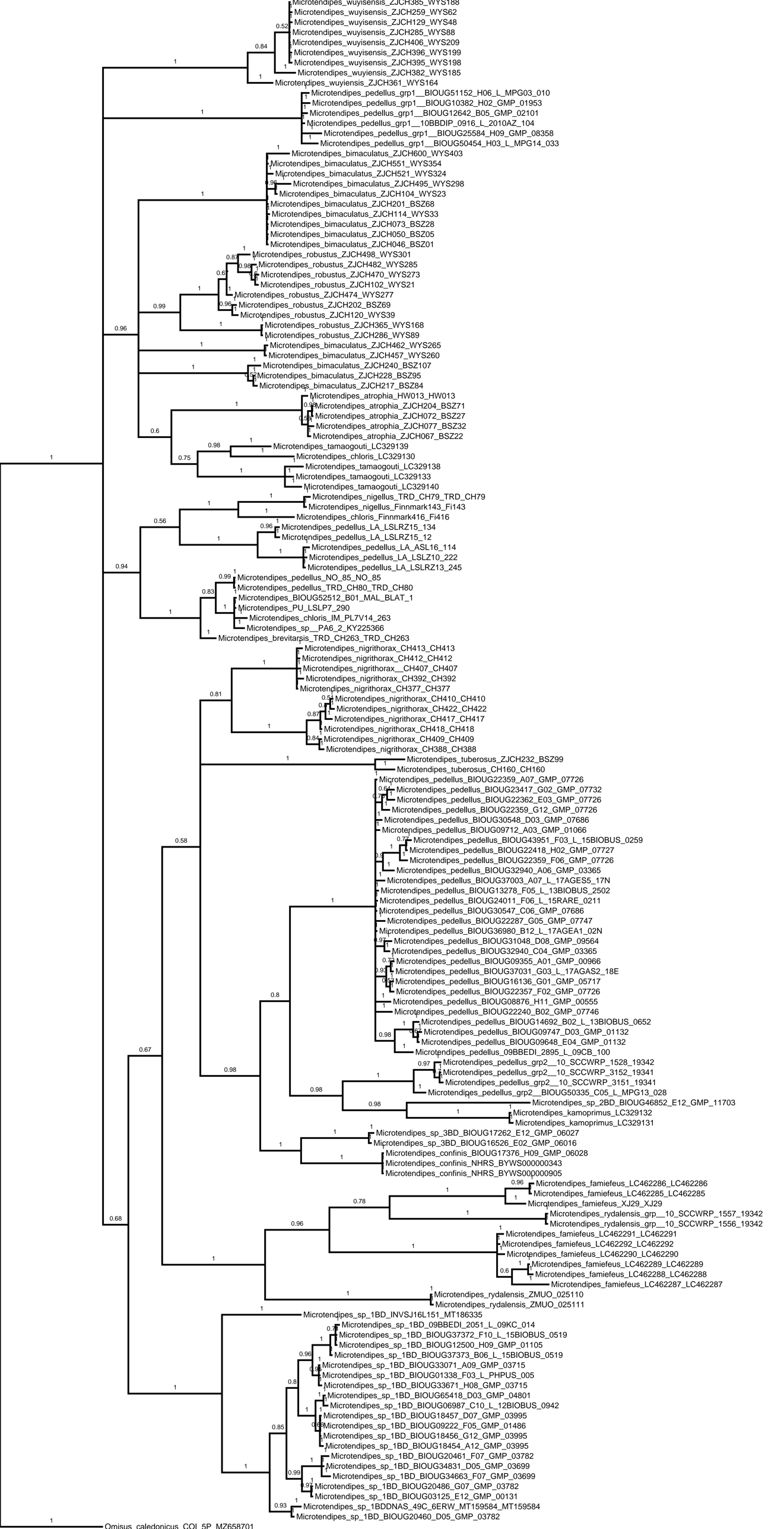

Supplement: Supplementary file 1 [file insects-14-00227-s001.zip › Figure S2.pdf]

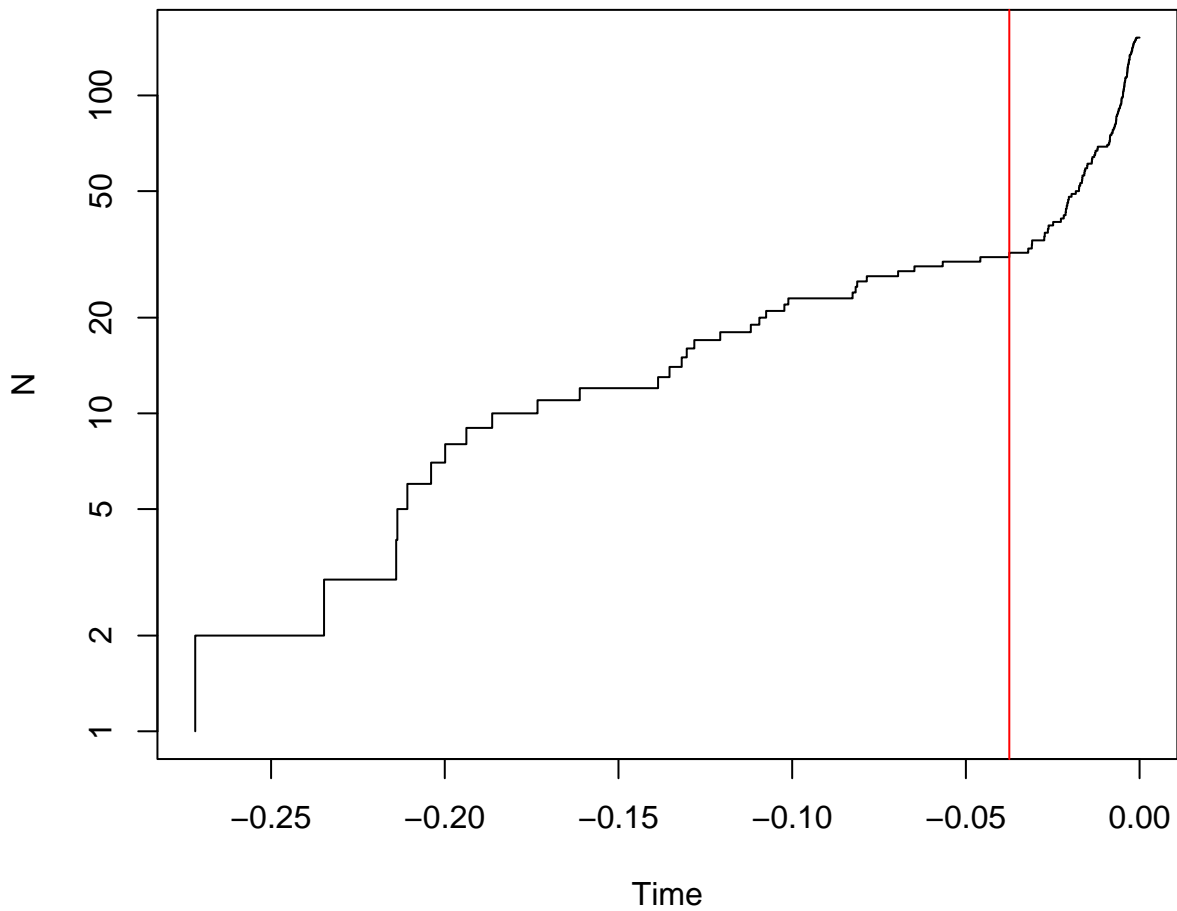

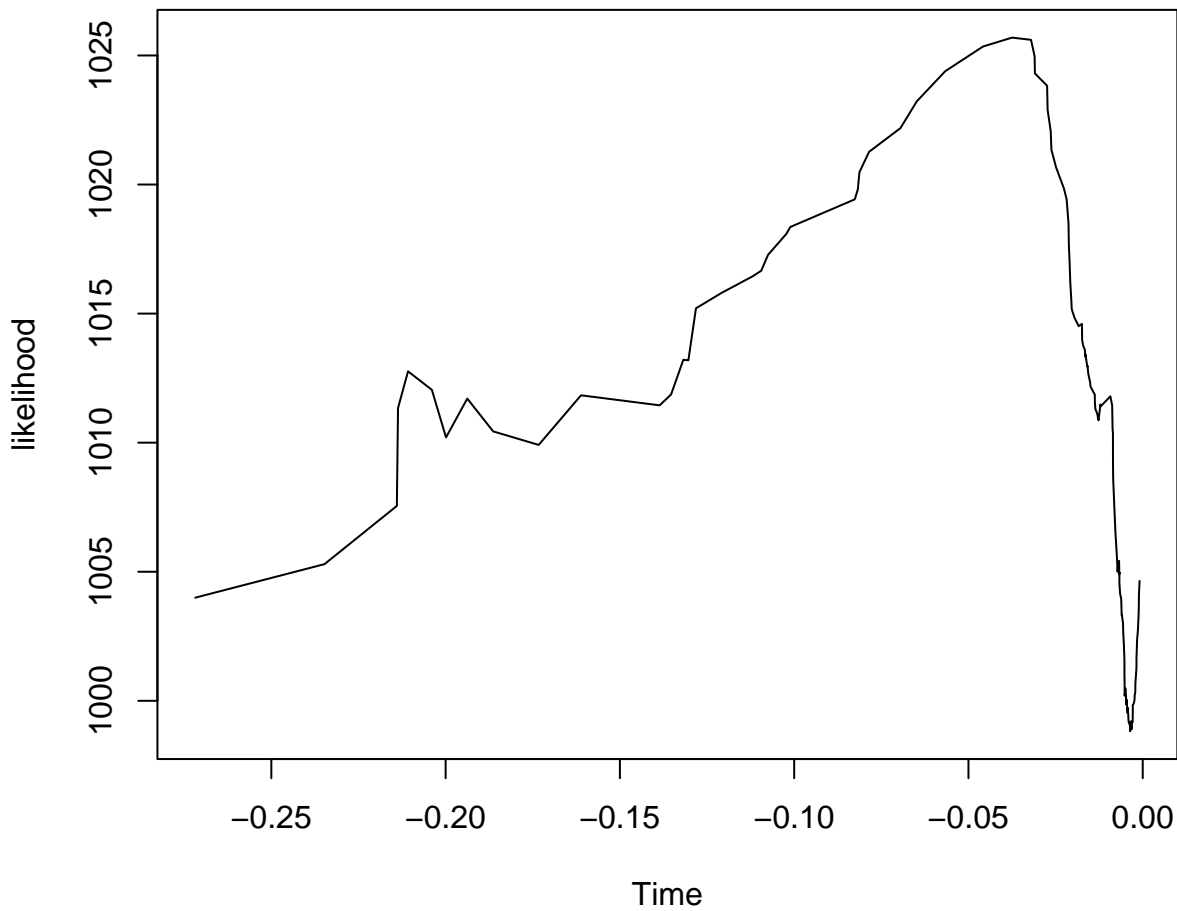

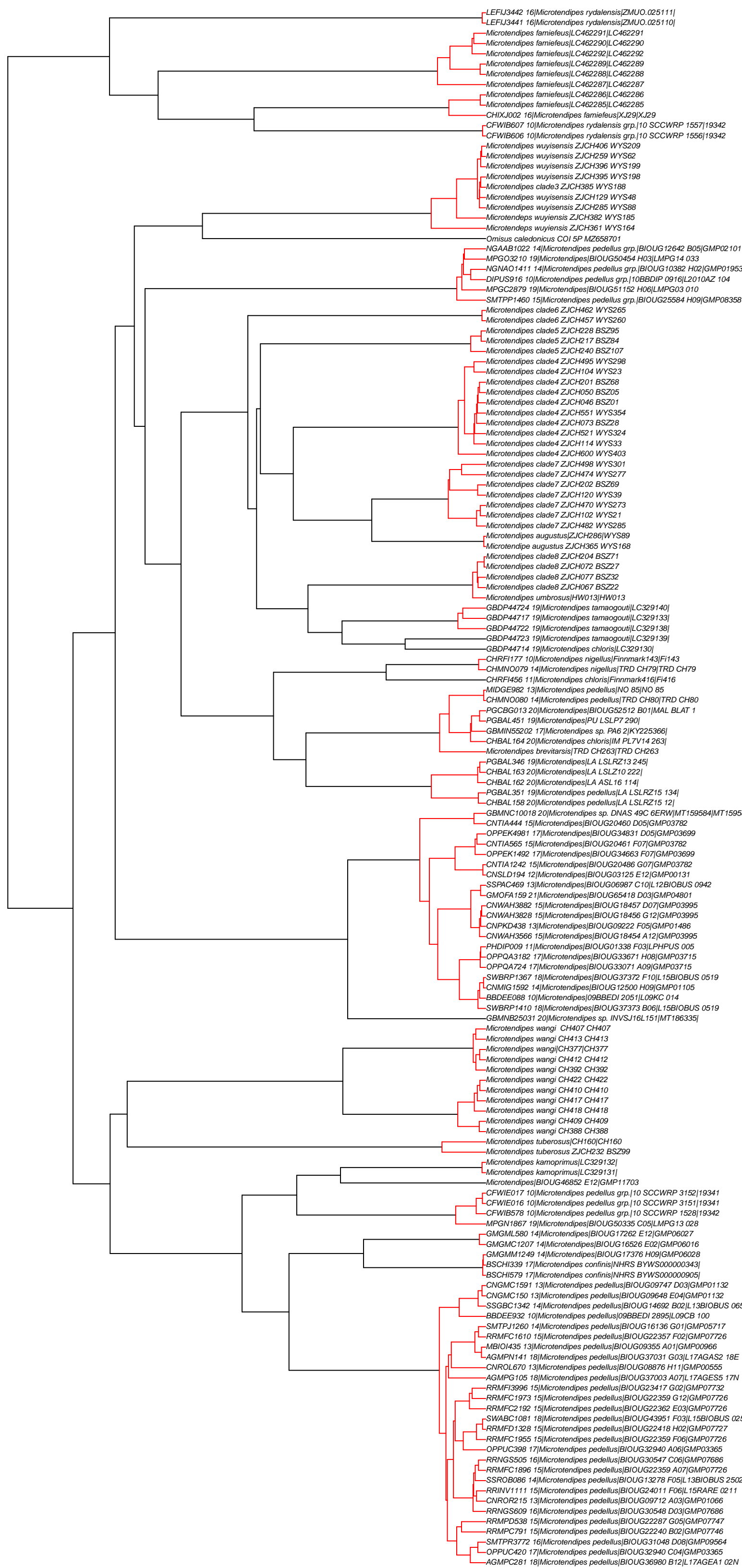

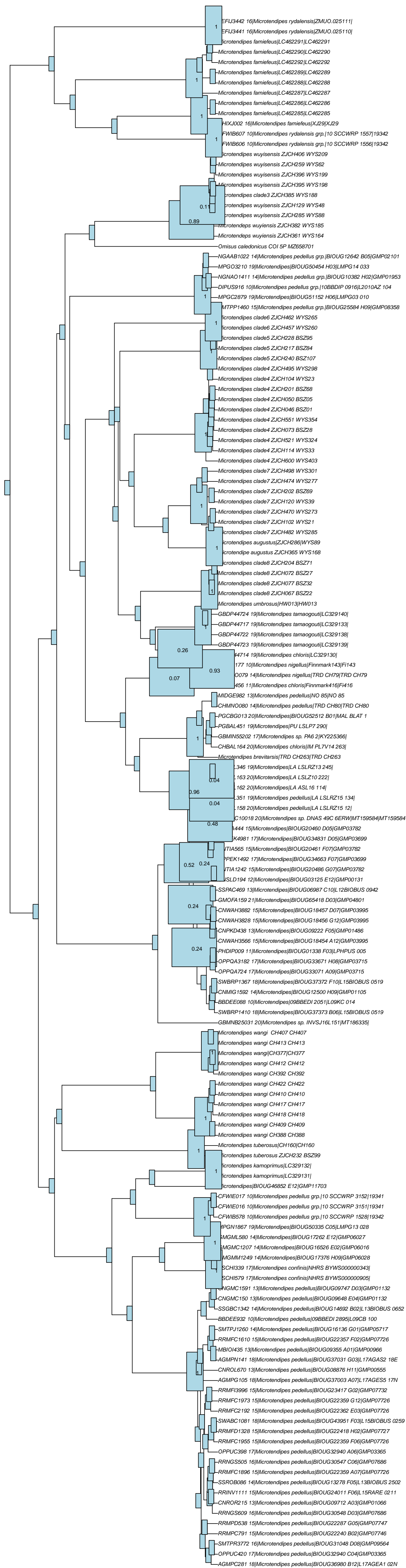

Supplement: Supplementary file 1 [file insects-14-00227-s001.zip › Figure S4.pdf]

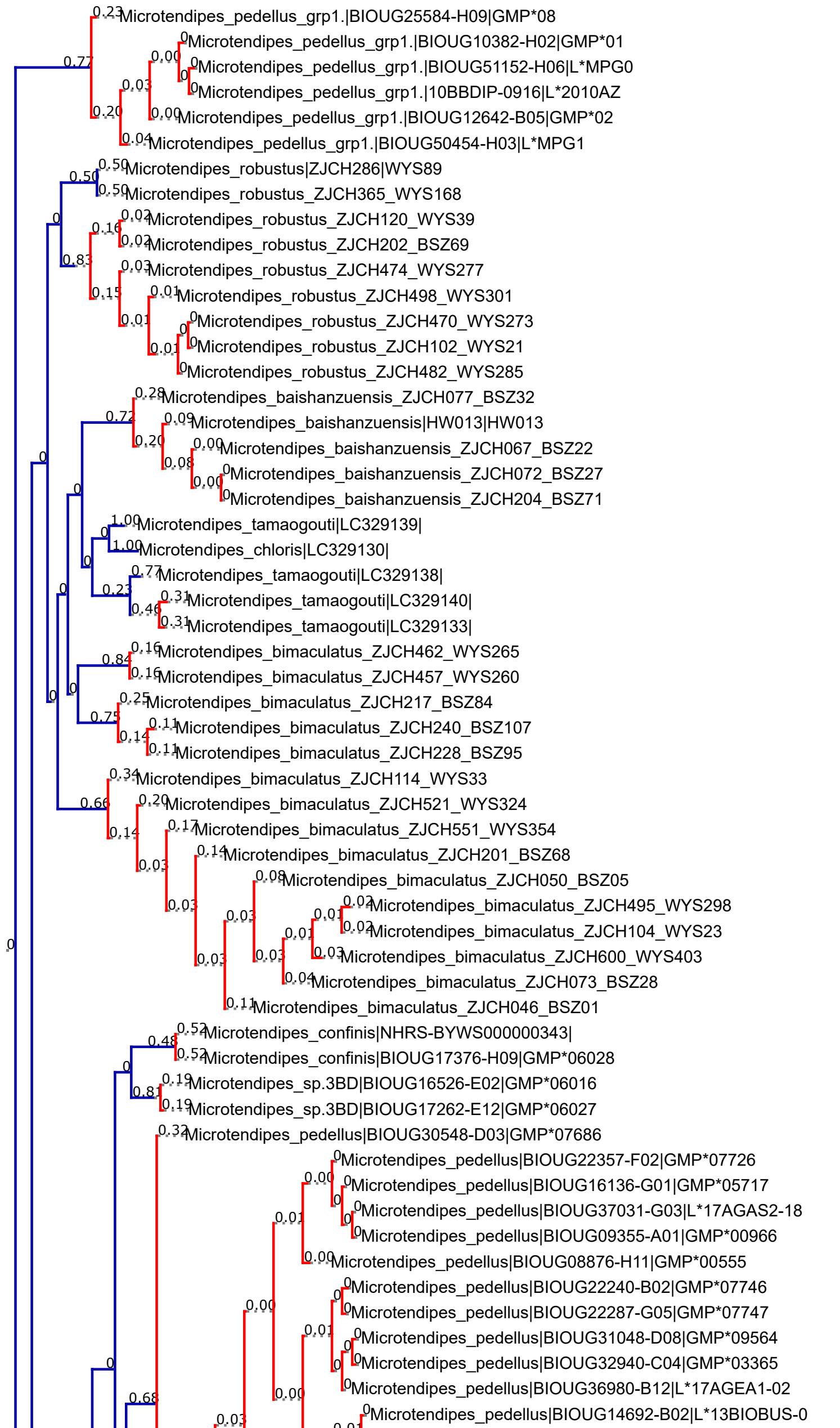

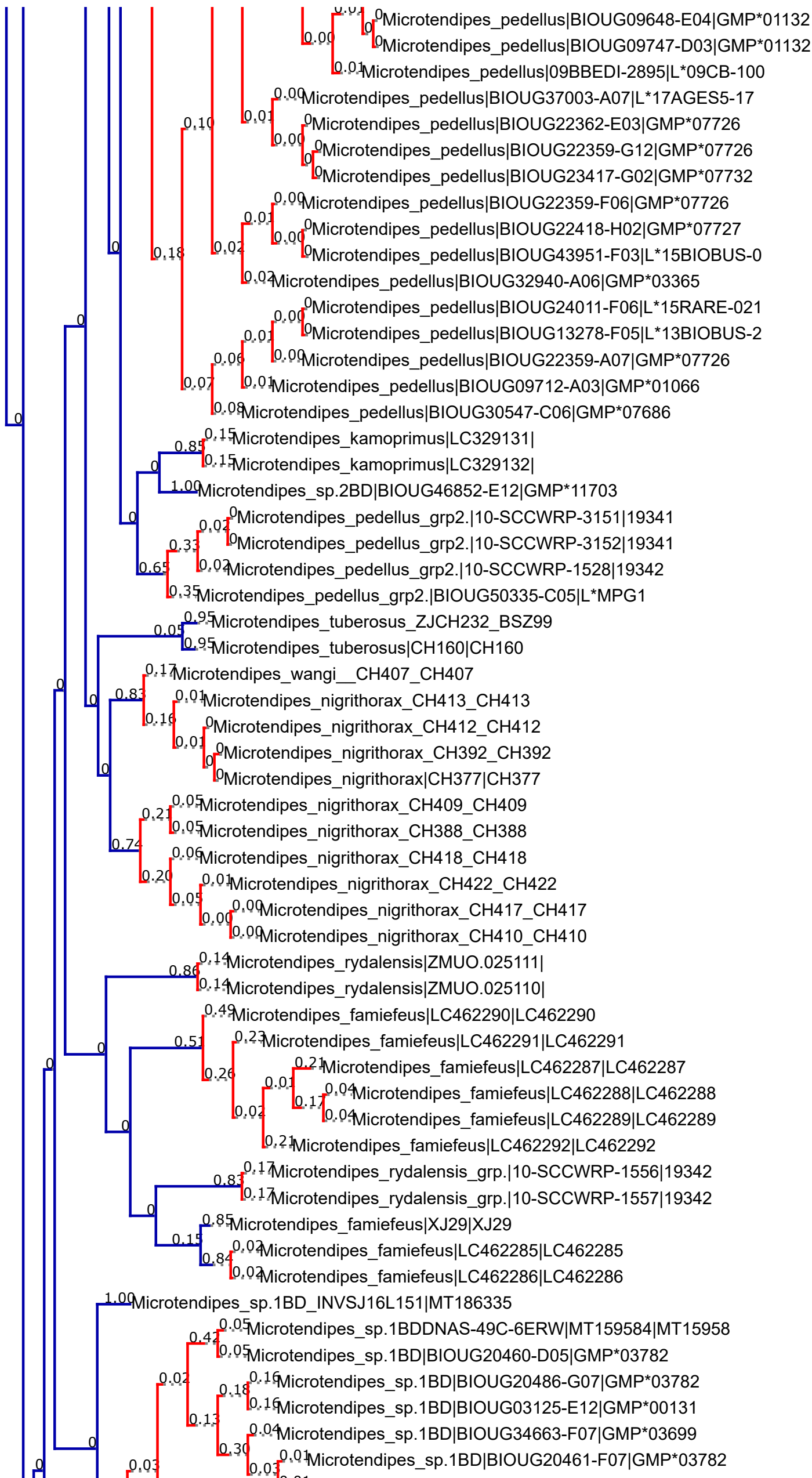

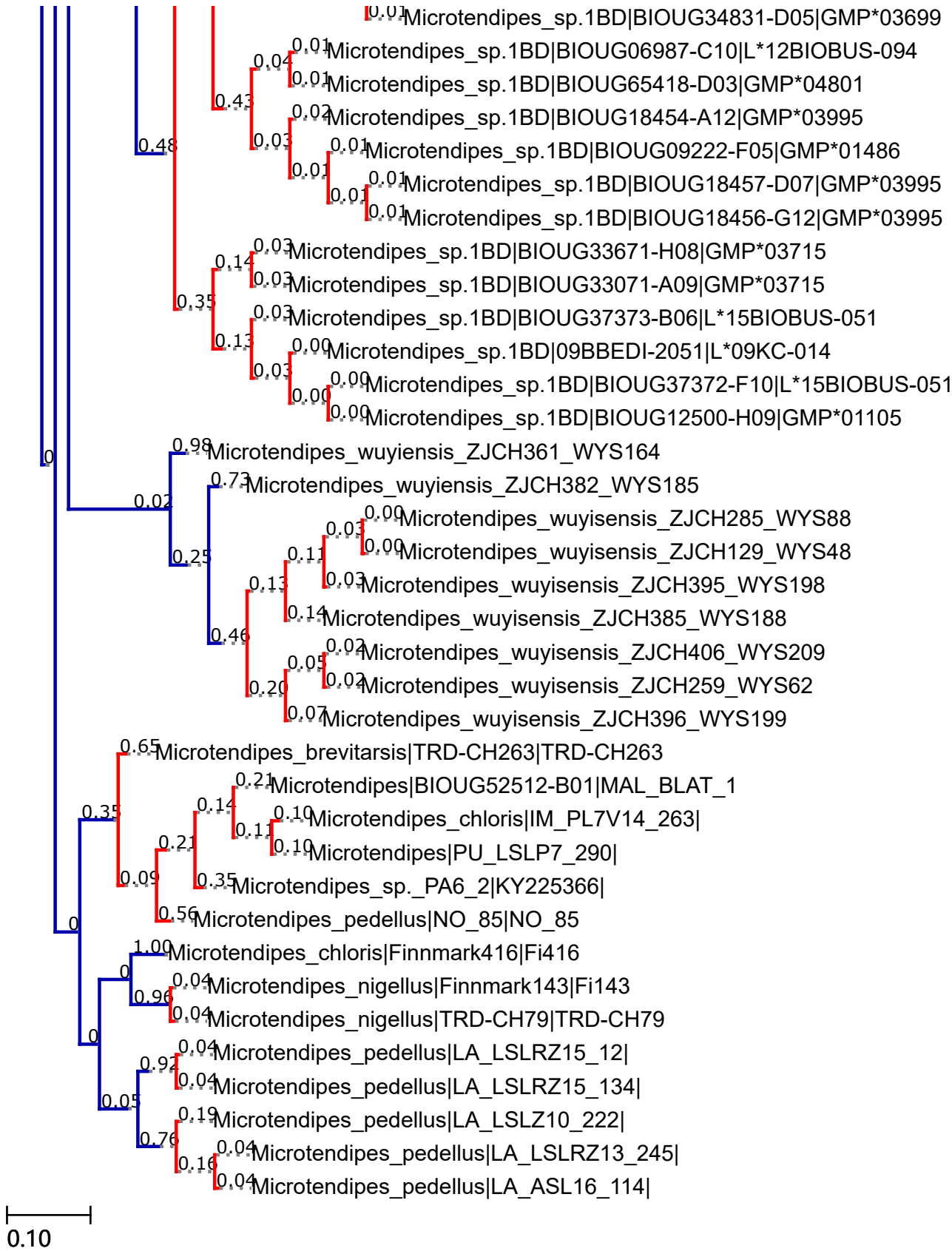

Supplement: Supplementary file 1 [file insects-14-00227-s001.zip › Figure S5.pdf]
